# Prevalence and Risk Factors of Asthma, Rhinitis, and Eczema and Their Multimorbidity among Young Adults in Kuwait: A Cross-Sectional Study

**DOI:** 10.1155/2017/2184193

**Published:** 2017-08-30

**Authors:** Ali H. Ziyab

**Affiliations:** Department of Community Medicine and Behavioral Sciences, Faculty of Medicine, Kuwait University, Kuwait, Kuwait

## Abstract

**Objective:**

To estimate the prevalence of allergic diseases and allergic multimorbidity (coexistence) among young adults in Kuwait and to examine associations between risk factors with allergic diseases and allergic multimorbidity.

**Methods:**

A cross-sectional study was conducted by enrolling 1,154 students, aged 18–26 years, attending Kuwait University. Participants self-completed a questionnaire on symptoms and clinical history of allergic diseases. Prevalence ratios (PRs) and 95% confidence intervals (CIs) were estimated by applying Poisson regression with robust variance estimation.

**Results:**

The prevalence of current asthma, rhinitis, and eczema was estimated to be 11.9% (135/1135), 20.4% (232/1138), and 9.2% (105/1143), respectively. The coexistence of “asthma and rhinitis” (5.1%, 57/1125) was the most frequent allergic multimorbidity. Both maternal history (PR = 3.97, 95% CI: 2.32–6.80) and paternal history (PR = 1.72, 95% CI: 1.10–2.68) of allergy were independently associated with having two or more coexisting allergic diseases. The joint effect of having both maternal and paternal history of allergy was associated with 8.16 times (95% CI: 4.19–15.90) higher risk of allergic multimorbidity.

**Conclusion:**

Allergic diseases and allergic multimorbidity are common among young adults in Kuwait and their burden mirrors that of westernized countries. Parental history of allergy is a strong predisposing factor for allergic multimorbidity.

## 1. Introduction

Allergic diseases, including asthma, rhinitis, and eczema, have emerged as a global public health challenge due to their elevated prevalence and the associated clinical and social burden [1, 2]. The International Study of Asthma and Allergies in Childhood (ISAAC) provided an unmatched opportunity to explore trends in the prevalence of allergic diseases and their risk factors over time in children [3]. Reports based on the ISAAC study revealed the existence of both between- and within-countries variations in the prevalence of allergic diseases [1, 4]. For instance, among children aged 13 to 14 years, the 12-month prevalence of eczema, asthma, and rhinitis symptoms ranged from 0.2% to 24.6% [5], from 3.4% to 31.2% [6], and from 4.5% to 45.1% [7], respectively. The disparity in the prevalence across nations and the changing trends over time between and within populations are indicators of the importance of environmental factors, in addition to the genetic elements, in disease pathogenesis.

Although allergic diseases can be thought of as single entities, their multimorbidity (i.e., coexistence/cooccurrence) is a common phenomenon. A meta-analysis estimated the worldwide prevalence of the coexistence of asthma, rhinitis, and eczema to be 1.17%, which is 9.8 times higher than what could be expected by chance alone [8]. In contrast, the “allergic march” concept suggests that allergic diseases develop sequentially with eczema, most often, being the index condition [9]. However, emerging evidence suggests that allergic diseases rather coexist than follow a progressive atopic march [10]. Results from the Swedish BAMSE birth cohort showed that the multimorbidity of allergic diseases is frequent during the first 12 years of life [11]. Similarly, results from the German Multicenter Allergy Study (MAS) indicate that allergic multimorbidity is common in childhood and persists up to early adulthood [12], hence indicating that allergic diseases share common developmental mechanisms and their natural history is complicated by their coexistence.

In Kuwait, the ISAAC study, which estimated the prevalence of allergic diseases among schoolchildren, was conducted in 1995-1996 [13] and in 2001-2002 [14]. The estimated prevalence of current symptoms of asthma, rhinitis, and eczema was 7.6%, 27.6%, and 8.3%, respectively, among schoolchildren aged 13 to 14 years in Kuwait [14]. These estimates indicate that allergic diseases are common among schoolchildren in Kuwait and are comparable to estimates from westernized countries [4]. Although the aforementioned studies provided valuable information on the burden of allergic diseases among schoolchildren, there remains a lack of empirical knowledge on the burden and risk factors of allergic diseases among young adults in Kuwait. In addition, it is important to understand how the prevalence of asthma, rhinitis, and eczema changes if youth cohorts mature and move into the age of young adults. Therefore, this cross-sectional study aimed to estimate the prevalence of allergic diseases and allergic multimorbidity among university students (young adults) in Kuwait. Moreover, associations between common risk factors with allergic diseases and allergic multimorbidity were examined.

## 2. Methods

### 2.1. Study Design and Participants

A cross-sectional study was conducted among students enrolled at Kuwait University (KU), which represents the only public university and the largest higher-education institution in Kuwait, enrolling approximately 36,000 undergraduate and graduate students with a female-to-male ratio of around 3 : 1. The current study enrolled 1,154 students during the period from January to May 2015. KU consists of 15 colleges scattered across five campuses. This study enrolled students from the five campuses of KU using proportional allocation to the size strategy through estimating the number of students (participants) needed from each campus relative to the entire population of students at KU. Hence, campuses with larger student body received more weight in the study sample size compared to smaller campuses, increasing the representativeness of the study sample. A random sample of instructors from each of the five campuses were emailed and asked about their willingness to allow the research team to introduce the study and distribute the questionnaire to students. Around 65% replied with positive responses. Students who were available at the selected classrooms were invited to participate; thus, study participants were recruited using convenience sampling. Students aged ≥ 18 years who were registered at the different colleges of KU at the time of the study were eligible to participate in the study. The study was approved by the Health Sciences Center Ethical Committee at KU. Written informed consent was obtained from all study participants. Upon consenting, study participants were asked to self-complete a questionnaire that included questions on sociodemographic and lifestyle factors and maternal history and paternal history of allergic diseases and adapted the core items from the ISAAC questionnaire regarding symptoms of asthma, rhinitis, and eczema [3].

### 2.2. Definitions of Allergic Diseases

A set of criteria based on the self-reported symptoms and previous clinical diagnosis/history were used to define asthma, rhinitis, and eczema in the last 12 months. The current (12-month) presence of asthma was defined by a positive response to “ever doctor-diagnosed asthma” and either “use of asthma medications in the last 12 months” or “wheezed in the last 12 months.” Current rhinitis was defined as “ever doctor-diagnosed rhinitis” plus “having problems with sneezing, runny, or blocked nose in the absence of cold or flu in the last 12 months.” Current eczema was defined as “ever doctor-diagnosed eczema” or “ever having itchy rash coming and going for at least six months” plus “having an itchy rash at any time in the last 12 months that affected the folds of the elbows, behind the knees, in front of the ankles, under the buttocks, or around the neck, ears, or eyes.”

### 2.3. Allergic Multimorbidity

Combinations of the three study-defined diseases (i.e., current asthma, rhinitis, and eczema) resulted in eight nonoverlapping groups of single and coexisting allergic diseases, namely, groups with “no allergic disease,” “asthma only,” “rhinitis only,” “eczema only,” “asthma + rhinitis,” “asthma + eczema,” “rhinitis + eczema,” and “asthma + rhinitis + eczema.” An alternative categorization, as the following, was also considered: “no allergic disease,” “one allergic disease,” and “two or more (≥2) allergic diseases.”

### 2.4. Covariates

Information on covariates (potential confounders) was obtained using the questionnaire. Study participants reported maternal history and paternal history of asthma, which were defined as a positive answer to the following question: “Has your mother/father ever been diagnosed with asthma by a doctor?” (separate questions were asked to inquire about mother's and father's asthma status). Information on parental history of eczema and rhinitis was collected in a similar manner. If one or more of these allergic conditions (i.e., asthma, rhinitis, or eczema) were present in parents, they were regarded as having a “history of allergic disease.” Moreover, we collected information on smoking habits (ever cigarettes and/or water pipe/hookah smoking), regular exposure to environmental tobacco smoke (ETS) inside and/or outside the house (i.e., exposure to cigarettes and/or water pipe/hookah smoke), birth order (first, second, and third or more), and mode of birth (vaginal or Caesarean section). Study participants also self-reported having a cat or a dog at home during the past 12 months (in the past 12 months, have you had a cat/dog in your home?).

### 2.5. Statistical Analysis

Descriptive analyses were conducted to estimate frequencies and proportions of categorical variables and medians and 5th and 95th percentiles of continuous variables. The 12-month prevalence and proportions of symptoms and multimorbidity (coexistence) of asthma, rhinitis, and eczema were estimated. Sex-based differences in proportions of allergic diseases were assessed using chi-square (*χ*^2^) tests.

Associations between risk factors with asthma, rhinitis, and eczema and their multimorbidity were evaluated. For each outcome variable, we tested two regression models, an unadjusted (crude) model and an adjusted model. To control for the effect of potential confounders, risk factors that demonstrated possible association with the outcomes in the unadjusted models (i.e.,* P* value ≤ 0.2, as suggested by Maldonado and Greenland [15]) were simultaneously entered into the multivariable regression models. In additional analysis, associations between having both maternal history and paternal history of allergic disease and having both a cat and a dog at home in the past 12 months with allergic diseases and allergic multimorbidity were assessed. Regardless of statistical significance, sex and age were included as potential confounders in all multivariable regression models. The prevalence of allergic diseases is not a rare event (i.e., >10%); hence, odds ratios are likely to overestimate relative risks [16]. Therefore, to directly estimate crude and adjusted prevalence ratios (PRs) and their 95% confidence intervals (CIs), we applied the modified Poisson regression with robust variance estimation using the GENMOD procedure in SAS 9.4 (SAS Institute, Cary, North Carolina, USA) [17, 18]. All statistical analyses were conducted using SAS 9.4. The statistical significance level was set to *α* = 0.05 for all association analyses.

## 3. Results

### 3.1. Characteristics of Study Participants

The current study enrolled 1,154 students out of the 1,561 students who were approached (response proportion: 73.9%). Of the total study sample, 892 were females and 262 were males (Table 1). The majority of the study participants were aged between 18.0 and 26.0 years, with a median age of 20.0 years. Ever tobacco smoking (i.e., cigarette and/or water pipe/hookah) was reported by 14.2% (163/1,151) of the study sample, with 51.9% (136/262) of males compared to 3.0% (27/889) of females being ever smokers (Table 1).

### 3.2. Prevalence of Symptoms of Allergic Diseases

With regard to asthma symptoms, the lifetime (ever) prevalence of wheezing was 22.5% (95% CI: 20.1–25.1), which was more frequent in male (29.0%) than in female (20.6%) participants (*P* = 0.005; Table 2). Similarly, the 12-month prevalence of wheezing was higher among males (19.2%) than females (13.2%;* P* = 0.018). The prevalence of self-reported ever doctor-diagnosed asthma was 19.1% (95% CI: 16.8–21.5), with more males being affected than females (26.1% versus 17.0%;* P* = 0.001). Hence, in general, symptoms of asthma were more prevalent among male than female study participants.

The lifetime and 12-month prevalence of rhinitis symptoms (i.e., sneezing or runny or blocked nose in the absence of cold or flu) were 43.1% (95% CI: 40.1–46.0) and 37.7% (95% CI: 34.8–40.6), respectively (Table 2). Symptoms of allergic conjunctivitis (i.e., itchy eyes associated with nasal problems) in the last 12 months affected 21.2% (95% CI: 18.8–23.8) of study participants. Ever doctor-diagnosed rhinitis was reported by 30.5% (95% CI: 27.8–33.3) of the total study sample. There were no statistically significant sex-based differences in all reported symptoms of rhinitis (Table 2).

The prevalence of ever doctor-diagnosed eczema was 20.2% (95% CI: 17.9–22.7), which was reported more frequently by female (23.0%) than male (10.8%) participants (*P* < 0.001; Table 2). Also, more female (24.3%) than male (17.2%) students reported itchy rash in the last 12 months (*P* = 0.002). Although the proportion of female compared to male participants reporting itchy rash affecting flexural area was higher (13.6% versus 9.3%), this difference did not gain statistical significance (*P* = 0.068; Table 2).

### 3.3. Prevalence of Study-Defined Current Asthma, Rhinitis, and Eczema

The 12-month (current) prevalence of allergic diseases was estimated using a set of criteria based on self-reported symptoms and history of clinical diagnosis. The 12-month prevalence of asthma was 11.9% (95% CI: 10.1–13.9), which showed a trend to be higher among male (15.2%) than female (10.9%) participants (*P* = 0.065; Table 2). Rhinitis affected 20.4% (95% CI: 18.1–22.9) of the study population in the last 12 months with no sex-based differences. The 12-month prevalence of eczema (9.2%, 95% CI: 7.6–11.0) was statistically significantly higher among female compared to male study participants (10.2% versus 5.8%;* P* = 0.029; Table 2).

### 3.4. Prevalence of Allergic Multimorbidity

Based on the study-defined current asthma, rhinitis, and eczema, eight nonoverlapping categories of allergic diseases were established. Asthma only in the last 12 months was more prevalent among males compared to females (8.6% versus 4.7%;* P* = 0.046; Table 3), whereas more female than male students reported eczema only in the last 12 months (7.3% versus 3.5%;* P* = 0.036). The coexistence of “asthma and rhinitis” was the most frequent allergic multimorbidity (5.1%, 95% CI: 3.9–6.5; Table 3). The coexistence of “asthma, rhinitis, and eczema” was evident in 0.6% (95% CI: 0.3–1.3) of the total study sample. There were no statistically significant sex-based differences in the prevalence of allergic multimorbidity. Moreover, the prevalence of having any one allergic disease and that of having two or more (≥2) allergic diseases were 25.3% (95% CI: 22.8–28.0) and 7.9% (95% CI: 6.4–9.7), respectively, with no sex-related differences (Table 3).

### 3.5. Associations between Common Risk Factors and Allergic Diseases

Associations (crude and adjusted) between common risk factors with current asthma, rhinitis, and eczema were assessed (Table 4). In the adjusted analysis, ever smoking, regular exposure to ETS, current exposure to cat and dog at home, and maternal history of allergic disease were statistically significantly associated with current asthma. Participants with maternal history of allergic disease had 3.15 times (95% CI: 2.10–4.71) higher prevalence of asthma compared to participants with nonallergic mothers (Table 4). Moreover, ever smoking was associated with higher prevalence of current asthma (PR = 1.70, 95% CI: 1.11–2.61). On the other hand, results of adjusted analysis showed that current exposure to cat (PR = 1.42, 95% CI: 1.08–1.87) and maternal history (PR = 1.82, 95% CI: 1.39–2.39) and paternal history (PR = 1.47, 95% CI: 1.14–1.89) of allergic disease were associated with higher prevalence of current rhinitis (Table 4). With regard to eczema, only maternal history (PR = 1.62, 95% CI: 1.05–2.49) and paternal history (PR = 1.54, 95% CI: 1.02–2.33) of allergic disease were associated with higher prevalence of current eczema in the adjusted analysis (Table 4).

### 3.6. Associations between Common Risk Factors and Allergic Multimorbidity

Associations between risk factors with having one allergic disease and having two or more (≥2) allergic diseases were explored (Table 5). In adjusted models, participants with maternal history and paternal history of allergic disease were more likely to have single allergic disease as well as coexisting allergic diseases compared to participants with no parental history of allergic disease. For example, maternal history of allergic disease was associated with 3.97 times (95% CI: 2.32–6.80) higher prevalence of having ≥2 allergic diseases, whereas paternal history of allergic disease was associated with 1.72 times (95% CI: 1.10–2.68) higher prevalence of having ≥2 allergic diseases (Table 5). Moreover, exposure to ETS and current exposure to cat were associated with having ≥2 allergic diseases.

### 3.7. Associations between Parental History of Allergy and Exposure to Pets with Allergic Diseases and Multimorbidity

In further analysis, associations between having both maternal history and paternal history of allergic disease and having both cat and dog at home in the past 12 months with allergic diseases and allergic multimorbidity were assessed. Strong and statistically significant associations were found when study participants having both maternal history and paternal history of allergic disease were compared with participants who had neither (Table 6). For instance, maternal history and paternal history of allergy were associated with 8.16 times (95% CI: 4.19–15.90) higher risk of having ≥2 coexisting allergic diseases. Similarly, we found 3.73 times (95% CI: 1.96–7.12) higher risk of having ≥2 coexisting allergic diseases in participants with both a dog and a cat at home compared to participants without pets (Table 6).

## 4. Discussion

This cross-sectional study estimated, for the first time, the burden of allergic diseases, including asthma, rhinitis, and eczema, and their multimorbidity among university students (young adults) in Kuwait. Also, associations between common risk factors with allergic diseases and multimorbidity were explored. Results of this study showed that allergic diseases affect a considerable proportion of university students in Kuwait. The estimated 12-month prevalence of study-defined asthma, rhinitis, and eczema was 11.9%, 20.4%, and 9.2%, respectively. The coexistence of asthma and rhinitis (5.1%) was the most frequent allergic multimorbidity. Asthma, rhinitis, and eczema coexisted in 0.6% of the study sample. In general, the prevalence of allergic multimorbidity did not demonstrate sex-related disparities; however, the single occurrence of asthma was more common among male than female participants, whereas eczema occurred more frequently among females compared to males. Having parents with history of allergic disease was a strong predictor of reporting current allergic diseases and allergic multimorbidity. Moreover, having pets at home during the past 12 months was associated with higher risk of allergies. Therefore, this study demonstrates that allergic diseases and their multimorbidity are common among university students in Kuwait and parental history of allergy and having pets at home are strong risk factors.

The current study estimated the 12-month prevalence of wheezing to be 14.6%, which is similar to estimates from the 1995-1996 ISAAC study (16.1%) and almost double what was reported by the 2001-2002 ISAAC study (7.6%) in Kuwait [13, 14]. Moreover, the estimated 12-month prevalence of wheezing is in agreement with the global estimate of 14.1% [6] and with results of a Swedish study (16.1%) conducted among adults aged 20 to 44 years [19]. With regard to sex differences, the current study and the ISAAC studies conducted in Kuwait demonstrated that current wheezing is more prevalent among males compared to females. This finding contradicts the common observation of a sex reversal (switchover) in symptoms of asthma, which suggests that before puberty asthma symptoms predominantly affect boys, whereas girls are more likely to be affected during adolescence and adulthood [20–22]. Results of a study from Saudi Arabia, a neighboring country, conducted among 16-year-old to 18-year-old adolescents agreed with our results by showing that current wheezing is more prevalent among males (20.3%) compared to females (16.9%) [23]. Since ever tobacco smoking (males: 51.9% versus females: 3.0%) and exposure to ETS (males: 46.5% versus females: 30.7%) were differentially reported by the two sexes, we hypothesized that the observed sex differences in asthma symptoms might be explained by the disparity in smoking and ETS prevalence between the two sexes. However, results of the multivariable regression model, adjusting for ever tobacco smoking and regular exposure to ETS in addition to other potential confounders, showed that females are less likely to have current asthma compared to males (PR = 0.79,* P* = 0.301; Table 4), though this difference was not statistically significant. Hence, smoking and ETS do not seem to fully explain the observed higher prevalence of asthma symptoms among males compared to females. Therefore, future etiologic studies, incorporating lung function testing, are needed to further confirm this sex-based disparity in asthma symptoms and investigate the possible underlying mechanisms.

This study showed that a large proportion of participants (37.7%) reported current symptoms of rhinitis. This observation is in agreement with reports from Spain (38.9%) [24] and United Kingdom (35.8%) [25] and the previous two ISAAC studies conducted in Kuwait (1995-1996: 30.7%; 2001-2001: 27.6%) [13, 14]. Moreover, our estimated prevalence of study-defined current rhinitis (20.4%) is within the global range of rhinitis prevalence (i.e., 10% to 30%) in the general population [26]. Furthermore, our observation of no sex-related differences in symptoms of rhinitis is further supported by previous observations [25].

The lifetime prevalence of doctor-diagnosed eczema was estimated to be 20.2% in the current study, which is comparable to prior estimates from the British Isles (24.3%) [27]. Moreover, the prevalence of study-defined current eczema (9.2%) is comparable to estimates among adults in the United States (10.2%) and Italy (8.1%) [28, 29]. The observed sex-related differences in the prevalence of current eczema, in which more females (10.2%) than males (5.8%) are affected, are in agreement with the existing scientific knowledge [30, 31].

With regard to allergic multimorbidity, the coexistence of “asthma and rhinitis” (5.1%) was the most common allergic multimorbidity in the current study, which is supported by the “one airway, one disease” notion [32]. This was followed by the coexistence of “rhinitis and eczema” (1.5%). The prevalence of coexistence of “asthma, rhinitis, and eczema” in our study (0.6%) was similar to estimates of two European studies carried out in Italy (0.7%) and Sweden (1.1%) among adults [29, 33]. Although sex-related disparities were found for the single occurrences of allergic diseases, we did not find differences in the prevalence of allergic multimorbidity when comparing the two sexes. Such an observation was previously reported [11, 12].

The most common risk factors for the development of allergic diseases and allergic multimorbidity were parental history of allergic disease followed by pet ownership. Having both parents with history of allergic disease was associated with 8.16 times higher risk of having ≥2 allergic diseases. Although previous studies have noted parental history of allergy as a risk factor for allergic multimorbidity [11, 12], the current study further demonstrated that the joint effect of both maternal history and paternal history of allergic disease is a strong predictor of allergic multimorbidity in the offspring. Also, we showed that having both a cat and a dog at home increased the risk of having ≥2 allergic diseases by 3.73 times. On the other hand, ever tobacco smoking and regular exposure to ETS were independently associated with increased asthma risk, an association that has been confirmed by a large meta-analysis [34].

Studies estimating the burden of allergic diseases among children are well represented in the scientific literature; however, this study is among the limited number of studies conducted among young adults to estimate the prevalence of asthma, eczema, and rhinitis and their coexistence and is the first in Kuwait. Moreover, the use of the validated ISAAC core questionnaire and the stringent epidemiologic definitions, incorporating both common symptoms and clinical diagnosis, to determine allergic disease status is considered a major strength of our study, which also aimed to reduce misclassification of outcomes. Study participants were enrolled using a sample of convenience, which might not provide a study sample that well represents the target population of university students. However, to maximize representativeness, we have enrolled students from all five campuses of KU using proportional allocation to the size strategy.

Self-selection bias, agreement/refusal to participate that is related to the exposure and disease, could be a source of bias in cross-sectional studies. However, self-selection bias is not of a great concern in the current study, since 73.9% (1,154/1,561) of eligible students who we were invited agreed to participate in the current study. Moreover, the sex distribution found in the current study sample (77.3% females and 22.7% males) closely resembles the sex distribution found in the target population of KU student body (73.5% females and 26.5% males). Hence, there is no indication of sex-related self-selection bias in the current study. Therefore, in the current study, self-selection bias is of little concern due to the acceptable response proportion (73.9%) and the similar sex distribution between the study sample and target KU population. However, selection bias cannot be completely dismissed in any epidemiologic study. The generalizability of our findings is limited to university students due to the fact that the sex distribution among university students (female-to-male ratio of around 3 : 1) does not resemble the sex distribution in the total population of young adults in Kuwait (female-to-male ratio of around 1.04).

Moreover, information bias, specifically recall bias, cannot be excluded, since offspring have reported their parents' history of having allergic disease. For instance, individuals with allergic disease might be more aware of their parents' allergies than nonallergic individuals, which might lead to differential misclassification (recall bias) that can either overestimate or underestimate the measure of association. To determine whether misclassification is of great concern in the current study, we compared the magnitude of measure of association found in this study to results of previously published birth cohort study, in which information about maternal/paternal history of allergic disease was collected from the parents themselves. Results from the German MAS birth cohort showed that, at age of 20 years, 18.5% (95% CI: 15.0–22.5%) of all participants with allergic parents had allergic multimorbidity (≥2 allergic diseases) as compared to only 6.3% (95% CI: 4.3–9.0%) of those with nonallergic parents [12]. Thus, parental history of allergic disease associated with 2.94-fold increased risk of having allergic multimorbidity at age of 20 years. In the current study, in additional analysis, we estimated the association between parental (maternal/paternal) history of allergies and offspring risk of having allergic multimorbidity. Results of this analysis showed that parental history of allergic disease associated with 3.35-fold (95% CI: 1.97–5.71; data not shown) higher risk of having allergic multimorbidity. Thus, the risk conferred by parental history of allergic disease in the current study (PR = 3.35) is slightly higher than what was estimated in the German MAS birth cohort (PR = 2.94). This compassion indicates that the influence of misclassification, if present, on results of this study is minimal.

## 5. Conclusion

The current study demonstrated that allergic diseases and allergic multimorbidity are common among university students in Kuwait and their burden mirrors that of westernized countries. The observation of higher prevalence of asthma among males compared to females contradicts the current scientific literature, which needs further corroboration and, if warranted, further etiologic investigations. Sex-related differences were observed for the occurrence of single allergic entities; however, the occurrence of allergic multimorbidity was not influenced by sex. Although both maternal history and paternal history of allergy were independently associated with higher risk of allergic diseases and allergic multimorbidity, the joint effect of both maternal history and paternal history of allergy was associated with increased risk of allergic multimorbidity. Similarly, having both a cat and a dog at home was a strong predictor of allergic multimorbidity.

## Figures and Tables

**Table 1:** Characteristics of study participants.

	Total	Males	Females
Sex, % (*n*/total)			
Male	22.7 (262/1154)		
Female	77.3 (892/1154)		
Age in years, median (5th, 95th percentile)	20.0 (18.0, 26.0)	21.0 (19.0, 33.0)	20.0 (18.0, 23.0)
Ever tobacco smoking^*∗*^, % (*n*/total)			
Yes	14.2 (163/1151)	51.9 (136/262)	3.0 (27/889)
Environmental tobacco smoke^†^, % (*n*/total)			
Yes	34.3 (386/1126)	46.5 (118/254)	30.7 (268/872)
Birth order, % (*n*/total)			
First	23.5 (270/1147)	22.7 (59/260)	23.8 (221/887)
Second	19.9 (228/1147)	21.9 (57/260)	19.3 (171/887)
Third or more	56.6 (649/1147)	55.4 (144/260)	56.9 (505/887)
Mode of birth, % (*n*/total)			
Vaginal	87.8 (956/1089)	85.3 (198/232)	88.5 (758/857)
Cesarean section	12.2 (133/1089)	14.7 (34/232)	11.5 (99/857)

^*∗*^Included participants reporting ever smoking cigarettes and/or ever smoking water pipe (hookah). ^†^Defined as regular exposure to tobacco smoke inside and/or outside the house.

**Table 2:** Prevalence of symptoms of asthma, rhinitis, and eczema in total study sample, stratified by sex.

	Total		Males	Females	*P* value^*∗*^
	% (*n*/total)	95% CI	% (*n*/total)	% (*n*/total)	
*Asthma symptoms*					
Wheezing ever	22.5 (248/1101)	20.1–25.1	29.0 (74/255)	20.6 (174/846)	0.005
Wheezing in the last 12 months	14.6 (161/1101)	12.6–16.9	19.2 (49/255)	13.2 (112/846)	0.018
Exercise-induced wheezing in the last 12 months	17.7 (198/1117)	15.5–20.1	24.5 (62/253)	15.7 (136/864)	0.001
Ever doctor-diagnosed asthma	19.1 (216/1134)	16.8–21.5	26.1 (67/257)	17.0 (149/877)	0.001
*Rhinitis symptoms*					
Ever sneezing/runny/blocked nose (when there is no cold/flu)	43.1 (478/1110)	40.1–46.0	40.9 (104/254)	43.7 (374/856)	0.438
Sneezing/runny/blocked nose (when there is no cold/flu) in the last 12 months	37.7 (415/1102)	34.8–40.6	32.9 (83/252)	39.1 (332/850)	0.078
Itchy eyes associated with nasal problems in the last 12 months	21.2 (231/1089)	18.8–23.8	19.8 (49/248)	21.6 (182/841)	0.524
Ever doctor-diagnosed rhinitis	30.5 (345/1132)	27.8–33.3	27.3 (71/260)	31.4 (274/872)	0.206
*Eczema symptoms*					
Itchy rash in the last 12 months	22.7 (251/1107)	20.2–25.3	17.2 (44/256)	24.3 (207/851)	0.002
Ever itchy rash coming/going for at least 6 months	17.7 (199/1123)	15.5–20.1	13.1 (34/260)	19.1 (165/863)	0.025
Itchy rash affecting flexural areas	12.6 (140/1111)	10.7–14.7	9.3 (24/258)	13.6 (116/853)	0.068
Ever doctor-diagnosed eczema	20.2 (230/1138)	17.9–22.7	10.8 (28/259)	23.0 (202/879)	<0.001
*Study-defined current* ^†^					
Asthma	11.9 (135/1135)	10.1–13.9	15.2 (39/257)	10.9 (96/878)	0.065
Rhinitis	20.4 (232/1138)	18.1–22.9	18.0 (47/261)	21.1 (185/877)	0.277
Eczema	9.2 (105/1143)	7.6–11.0	5.8 (15/261)	10.2 (90/882)	0.029

CI: confidence interval. ^*∗*^*P* values comparing male and female participants. ^†^See “Methods” for detailed information on the criteria used to define current asthma, rhinitis, and eczema.

**Table 3:** Prevalence of study-defined current asthma, rhinitis, and eczema as single morbidity and multimorbidity stratified by sex.

Allergic disease	Total		Males	Females	*P* value^*∗*^
	(*n* = 1125)		(*n* = 256)	(*n* = 869)	
	% (*n*)	95% CI	% (*n*)	% (*n*)	
None	66.8 (751)	63.9–69.5	69.1 (177)	66.0 (574)	
Asthma only	5.6 (63)	4.3–7.1	8.6 (22)	4.7 (41)	0.046
Rhinitis only	13.3 (150)	11.4–15.5	10.5 (27)	14.2 (123)	0.164
Eczema only	6.4 (72)	5.0–8.0	3.5 (9)	7.3 (63)	0.036
Asthma + rhinitis	5.1 (57)	3.9–6.5	5.9 (15)	4.8 (42)	0.630
Asthma + eczema	0.7 (8)	0.3–1.4	0.4 (1)	0.8 (7)	0.689
Rhinitis + eczema	1.5 (17)	0.9–2.4	1.6 (4)	1.5 (13)	0.997
Asthma + rhinitis + eczema	0.6 (7)	0.3–1.3	0.4 (1)	0.7 (6)	0.848
One allergic disease^†^	25.3 (285)	22.8–28.0	22.7 (58)	26.1 (227)	0.270
≥2 allergic diseases^‡^	7.9 (89)	6.4–9.7	8.2 (21)	7.8 (68)	0.996

CI: confidence interval. ^*∗*^*P* values comparing male and female participants. In cells with frequency < 5, Fisher's exact test was used to obtain *P* values. ^†^Indicating that the participant has either asthma, rhinitis, or eczema. ^‡^Indicating that the participant has at least two allergic diseases.

**Table 4:** Crude and adjusted associations between risk factors and study-defined current asthma, rhinitis, and eczema.

Independent variables	Asthma		Rhinitis		Eczema	
	Crude PR	Adjusted PR	Crude PR	Adjusted PR	Crude PR	Adjusted PR
	(95% CI)	(95% CI)^*⁋*^	(95% CI)	(95% CI)^*⁋*^	(95% CI)	(95% CI)^*⁋*^
Age^*∗*^	1.01 (0.97–1.06)	0.99 (0.93–1.05)	1.02 (0.99–1.05)	1.04 (1.01–1.07)	0.99 (0.94–1.06)	1.02 (0.95–1.09)
*P* value	0.553	0.730	0.225	0.007	0.969	0.663
Sex						
Male	1.00 (Ref.)	1.00 (Ref.)	1.00 (Ref.)	1.00 (Ref.)	1.00 (Ref.)	1.00 (Ref.)
Female	0.72 (0.51–1.02)	0.79 (0.51–1.24)	1.17 (0.88–1.56)	1.26 (0.88–1.79)	1.78 (1.05–3.01)	1.63 (0.80–3.31)
*P* value	0.065	0.301	0.283	0.208	0.033	0.177
Smoking^†^						
Never	1.00 (Ref.)	1.00 (Ref.)	1.00 (Ref.)		1.00 (Ref.)	
Ever	1.92 (1.34–2.75)	1.70 (1.11–2.61)	0.99 (0.72–1.38)		0.78 (0.44–1.39)	
*P* value	<0.001	0.014	0.971		0.397	
ETS^‡^						
No	1.00 (Ref.)	1.00 (Ref.)	1.00 (Ref.)		1.00 (Ref.)	1.00 (Ref.)
Yes	1.48 (1.08–2.03)	1.45 (1.02–2.05)	1.10 (0.87–1.40)		1.57 (1.09–2.27)	1.37 (0.91–2.08)
*P* value	0.017	0.038	0.437		0.015	0.137
Birth order						
First	1.19 (0.82–1.74)		1.04 (0.78–1.38)		1.26 (0.82–1.93)	
Second	1.16 (0.77–1.73)		1.25 (0.94–1.66)		1.04 (0.64–1.69)	
Third or more	1.00 (Ref.)		1.00 (Ref.)		1.00 (Ref.)	
*P* value	0.601		0.334		0.597	
Mode of birth						
Vaginal	1.00 (Ref.)		1.00 (Ref.)		1.00 (Ref.)	1.00 (Ref.)
C-section	1.31 (0.84–2.03)		1.06 (0.74–1.50)		0.55 (0.26–1.16)	0.68 (0.32–1.43)
*P* value	0.230		0.756		0.116	0.307
Cat exposure^$^						
No	1.00 (Ref.)	1.00 (Ref.)	1.00 (Ref.)	1.00 (Ref.)	1.00 (Ref.)	
Yes	1.64 (1.14–2.36)	1.44 (1.02–2.18)	1.33 (1.01–1.76)	1.42 (1.08–1.87)	1.15 (0.72–1.84)	
*P* value	0.008	0.043	0.043	0.013	0.556	
Dog exposure^$^						
No	1.00 (Ref.)	1.00 (Ref.)	1.00 (Ref.)		1.00 (Ref.)	
Yes	1.97 (1.18–3.26)	1.62 (1.01–2.60)	1.21 (0.76–1.93)		1.14 (0.52–2.48)	
*P* value	0.009	0.046	0.429		0.746	
Maternal allergy^*£*^						
No	1.00 (Ref.)	1.00 (Ref.)	1.00 (Ref.)	1.00 (Ref.)	1.00 (Ref.)	1.00 (Ref.)
Yes	3.08 (2.14–4.42)	3.15 (2.10–4.71)	2.04 (1.59–2.60)	1.82 (1.39–2.39)	1.80 (1.22–2.65)	1.62 (1.05–2.49)
*P* value	<0.001	<0.001	<0.001	<0.001	0.003	0.028
Paternal allergy^*£*^						
No	1.00 (Ref.)	1.00 (Ref.)	1.00 (Ref.)	1.00 (Ref.)	1.00 (Ref.)	1.00 (Ref.)
Yes	1.76 (1.25–2.48)	1.29 (0.92–1.82)	1.74 (1.37–2.23)	1.47 (1.14–1.89)	1.87 (1.25–2.77)	1.54 (1.02–2.33)
*P* value	0.001	0.147	<0.001	0.003	0.002	0.042

PR: prevalence ratio; CI: confidence interval; Ref.: reference; ETS: environmental tobacco smoke; C-section: Cesarean section. ^*∗*^Age was modeled as a continuous variable. ^†^Included participants reporting ever smoking cigarettes and/or ever smoking water pipe (hookah). ^‡^Defined as regular exposure to tobacco smoke inside and/or outside the house. ^$^Defined as having a cat/dog in the home during the past 12 months. ^*£*^Mother/father having history of doctor-diagnosed asthma, eczema, or rhinitis. ^*⁋*^Variables that had a *P* value ≤ 0.2 in the crude model were included in the adjusted (multivariable) model, except for age and sex, which were included in all adjusted models.

**Table 5:** Crude and adjusted associations between risk factors and allergic multimorbidity.

Independent variables	Allergic multimorbidity^*¥*^			
	One allergic disease^#^		≥2 allergic diseases^§^	
	Crude PR	Adjusted PR	Crude PR	Adjusted PR
	(95% CI)	(95% CI)^*⁋*^	(95% CI)	(95% CI)^*⁋*^
Age^*∗*^	1.01 (0.98–1.04)	1.01 (0.98–1.05)	1.03 (0.98–1.08)	1.02 (0.97–1.06)
*P* value	0.665	0.394	0.212	0.596
Sex				
Male	1.00 (Ref.)	1.00 (Ref.)	1.00 (Ref.)	1.00 (Ref.)
Female	1.15 (0.90–1.47)	1.06 (0.80–1.40)	1.00 (0.63–1.59)	1.35 (0.65–2.80)
*P* value	0.277	0.700	0.996	0.426
Smoking^†^				
Never	1.00 (Ref.)		1.00 (Ref.)	1.00 (Ref.)
Ever	0.90 (0.66–1.22)		1.69 (1.07–2.68)	1.65 (0.79–3.42)
*P* value	0.505		0.025	0.180
ETS^‡^				
No	1.00 (Ref.)		1.00 (Ref.)	1.00 (Ref.)
Yes	1.04 (0.85–1.29)		1.94 (1.31–2.86)	1.75 (1.14–2.69)
*P* value	0.688		<0.001	0.011
Birth order				
First	1.03 (0.81–1.33)		1.39 (0.89–2.19)	
Second	1.24 (0.98–1.58)		1.24 (0.74–2.08)	
Third or more	1.00 (Ref.)		1.00 (Ref.)	
*P* value	0.236		0.359	
Mode of birth				
Vaginal	1.00 (Ref.)		1.00 (Ref.)	
C-section	1.03 (0.76–1.39)		0.99 (0.53–1.85)	
*P* value	0.868		0.975	
Cat exposure^$^				
No	1.00 (Ref.)	1.00 (Ref.)	1.00 (Ref.)	1.00 (Ref.)
Yes	1.21 (0.94–1.55)	1.27 (0.99–1.62)	2.02 (1.31–3.10)	1.79 (1.11–2.90)
*P* value	0.149	0.061	0.001	0.018
Dog exposure^$^				
No	1.00 (Ref.)		1.00 (Ref.)	1.00 (Ref.)
Yes	1.19 (0.79–1.81)		1.91 (1.01–3.65)	1.07 (0.49–2.34)
*P* value	0.402		0.048	0.860
Maternal allergy^*£*^				
No	1.00 (Ref.)	1.00 (Ref.)	1.00 (Ref.)	1.00 (Ref.)
Yes	1.92 (1.56–2.36)	1.76 (1.41–2.20)	3.99 (2.52–6.32)	3.97 (2.32–6.80)
*P* value	<0.001	<0.001	<0.001	<0.001
Paternal allergy^*£*^				
No	1.00 (Ref.)	1.00 (Ref.)	1.00 (Ref.)	1.00 (Ref.)
Yes	1.75 (1.43–2.15)	1.48 (1.20–1.82)	2.45 (1.58–3.78)	1.72 (1.10–2.68)
*P* value	<0.001	<0.001	<0.001	0.017

PR: prevalence ratio; CI: confidence interval; Ref.: reference; ETS: environmental tobacco smoke; C-section: Cesarean section. ^*¥*^The “no allergic disease” category formed the common reference category. ^#^Indicating that the participant has either asthma, rhinitis, or eczema. ^§^Indicating that the participant has at least two allergic diseases. ^*∗*^Age was modeled as a continuous variable. ^†^Included participants reporting ever smoking cigarettes and/or ever smoking water pipe (hookah). ^‡^Defined as regular exposure to tobacco smoke inside and/or outside the house. ^$^Defined as having a cat/dog in the home during the past 12 months. ^*£*^Mother/father having history of doctor-diagnosed asthma, eczema, or rhinitis. ^*⁋*^Variables that had a *P* value ≤ 0.2 in the crude model were included in the adjusted (multivariable) model, except for age and sex, which were included in all adjusted models.

**Table 6:** Associations between having both maternal history and paternal history of allergy and having both cat and dog currently in the home with study-defined current asthma, rhinitis, and eczema and allergic multimorbidity.

Independent variables	Asthma	Rhinitis	Eczema	One allergic disease^#^	≥2 allergic diseases^§^
	Adjusted PR	Adjusted PR	Adjusted PR	Adjusted PR	Adjusted PR
	(95% CI)^*⁋*^	(95% CI)^*⁋*^	(95% CI)^*⁋*^	(95% CI)^*⁋*^	(95% CI)^*⁋*^
Maternal history and paternal history of allergy^†^					
No	1.00 (Ref.)	1.00 (Ref.)	1.00 (Ref.)	1.00 (Ref.)	1.00 (Ref.)
Yes	4.57 (2.81–7.43)	2.82 (2.02–3.92)	2.83 (1.71–4.69)	2.70 (2.08–3.50)	8.16 (4.19–15.90)
*P* value	<0.001	<0.001	<0.001	<0.001	<0.001
Current exposure to cat and dog^‡^					
No	1.00 (Ref.)	1.00 (Ref.)	1.00 (Ref.)	1.00 (Ref.)	1.00 (Ref.)
Yes	2.86 (1.55–5.27)	1.77 (1.03–3.04)	1.32 (0.45–3.85)	1.21 (0.62–2.35)	3.73 (1.96–7.12)
*P* value	<0.001	0.038	0.613	0.582	<0.001

PR: prevalence ratio; CI: confidence interval; Ref.: reference. ^#^Indicating that the participant has either asthma, rhinitis, or eczema. ^§^Indicating that the participant has at least two allergic diseases. ^*⁋*^Adjusted for sex and age. ^†^Both mother and father having history of doctor-diagnosed asthma, eczema, or rhinitis. Participants with both maternal history and paternal history of allergy were compared to those with neither. ^‡^Having both a cat and a dog at home during the past 12 months. Participants having both a cat and a dog at home were compared with those who had neither.

## References

[1] Asher M. I., Montefort S., Björkstén B. (2006). Worldwide time trends in the prevalence of symptoms of asthma, allergic rhinoconjunctivitis, and eczema in childhood: ISAAC Phases One and Three repeat multicountry cross-sectional surveys. *The Lancet*.

[2] Pawankar R., Canonica G. W., Holgate S. T., Lockey R. F. (2012). Allergic diseases and asthma: a major global health concern. *Current Opinion in Allergy and Clinical Immunology*.

[3] Asher M. I., Keil U., Anderson H. R. (1995). International study of asthma and allergies in childhood (ISAAC): rationale and methods. *European Respiratory Journal*.

[4] Mallol J., Crane J., von Mutius E., Odhiambo J., Keil U., Stewart A. (2013). The International Study of Asthma and Allergies in Childhood (ISAAC) Phase Three: a global synthesis. *Allergologia et Immunopathologia*.

[5] Odhiambo J. A., Williams H. C., Clayton T. O., Robertson C. F., Asher M. I. (2009). Global variations in prevalence of eczema symptoms in children from ISAAC Phase Three. *Journal of Allergy and Clinical Immunology*.

[6] Lai C. K. W., Beasley R., Crane J. (2009). Global variation in the prevalence and severity of asthma symptoms: phase three of the International Study of Asthma and Allergies in Childhood (ISAAC). *Thorax*.

[7] Aït-Khaled N., Pearce N., Anderson H. R., Ellwood P., Montefort S., Shah J. (2009). Global map of the prevalence of symptoms of rhinoconjunctivitis in children: The International Study of Asthma and Allergies in Childhood (ISAAC) Phase Three. *Allergy*.

[8] Pol D. H. J., Wartna J. B., Van Alphen E. I. (2015). Interrelationships between atopic disorders in children: A meta-analysis based on ISAAC questionnaires. *PLoS ONE*.

[9] Zheng T., Yu J., Oh M. H., Zhu Z. (2011). The atopic march: progression from atopic dermatitis to allergic rhinitis and asthma. *Allergy, Asthma & Immunology Research*.

[10] Belgrave D. C., Granell R., Simpson A. (2014). Developmental Profiles of Eczema, Wheeze, and Rhinitis: Two Population-Based Birth Cohort Studies. *PLoS Medicine*.

[11] Ballardini N., Kull I., Lind T. (2012). Development and comorbidity of eczema, asthma and rhinitis to age 12 - Data from the BAMSE birth cohort. *Allergy: European Journal of Allergy and Clinical Immunology*.

[12] Gough H., Grabenhenrich L., Reich A. (2015). Allergic multimorbidity of asthma, rhinitis and eczema over 20 years in the German birth cohort MAS. *Pediatric Allergy and Immunology*.

[13] Behbehani N. A., Abal A., Syabbalo N., Azeem A. A., Shareef E., Al-Momen J. (2000). Prevalence of asthma, allergic rhinitis, and eczema in 13- to 14-year-old children in Kuwait: an ISAAC study. *Annals of Allergy, Asthma & Immunology*.

[14] Owayed A., Behbehani N., Al-Momen J. (2008). Changing prevalence of asthma and allergic diseases among Kuwaiti children: An ISAAC study (phase III). *Medical Principles and Practice*.

[15] Maldonado G., Greenland S. (1993). Simulation study of confounder-selection strategies. *The American Journal of Epidemiology*.

[16] Zhang J., Yu K. F. (1998). What's the relative risk? A method of correcting the odds ratio in cohort studies of common outcomes. *Journal of the American Medical Association*.

[17] Spiegelman D., Hertzmark E. (2005). Easy SAS calculations for risk or prevalence ratios and differences. *The American Journal of Epidemiology*.

[18] Zou G. (2004). A modified poisson regression approach to prospective studies with binary data. *American Journal of Epidemiology*.

[19] Bjerg A., Ekerljung L., Middelveld R. (2011). Increased Prevalence of Symptoms of Rhinitis but Not of Asthma between 1990 and 2008 in Swedish Adults: Comparisons of the ECRHS and GA2LEN Surveys. *PLoS ONE*.

[20] Soto-Ramírez N., Ziyab A. H., Karmaus W. (2013). Epidemiologic methods of assessing asthma and wheezing episodes in longitudinal studies: Measures of change and stability. *Journal of Epidemiology*.

[21] Vink N. M., Postma D. S., Schouten J. P., Rosmalen J. G. M., Boezen H. M. (2010). Gender differences in asthma development and remission during transition through puberty: The TRacking Adolescents' Individual Lives Survey (TRAILS) study. *Journal of Allergy and Clinical Immunology*.

[22] Arathimos R., Granell R., Henderson J., Relton C. L., Tilling K., Lee Y. L. (2017). Sex discordance in asthma and wheeze prevalence in two longitudinal cohorts. *PLOS ONE*.

[23] Al Ghobain M. O., Al-Hajjaj M. S., Al Moamary M. S. (2012). Asthma prevalence among 16- to 18-year-old adolescents in Saudi Arabia using the ISAAC questionnaire. *BMC Public Health*.

[24] Batlles-Garrido J., Torres-Borrego J., Rubí-Ruiz T. (2010). Prevalence and factors linked to allergic rhinitis in 10 and 11-year-old children in Almería. Isaac Phase II, Spain. *Allergologia et Immunopathologia*.

[25] Kurukulaaratchy R. J., Karmaus W., Raza A., Matthews S., Roberts G., Arshad S. H. (2011). The influence of gender and atopy on the natural history of rhinitis in the first 18 years of life. *Clinical and Experimental Allergy*.

[26] Mucci T., Govindaraj S., Tversky J. (2011). Allergic Rhinitis. *Mount Sinai Journal of Medicine: A Journal of Translational and Personalized Medicine*.

[27] Anderson H. R. (2004). Trends in prevalence of symptoms of asthma, hay fever, and eczema in 12-14 year olds in the British Isles, 1995-2002: questionnaire survey. *BMJ*.

[28] Silverberg J. I., Hanifin J. M. (2013). Adult eczema prevalence and associations with asthma and other health and demographic factors: a US population-based study. *Journal of Allergy and Clinical Immunology*.

[29] Pesce G., Marcon A., Carosso A. (2015). Adult eczema in Italy: prevalence and associations with environmental factors. *Journal of the European Academy of Dermatology and Venereology*.

[30] Ziyab A. H., Raza A., Karmaus W. (2010). Trends in eczema in the first 18 years of life: Results from the Isle of Wight 1989 birth cohort study. *Clinical and Experimental Allergy*.

[31] Burr M., Dunstan F., Hand S., Ingram J., Jones K. (2013). The natural history of eczema from birth to adult life: a cohort study. *British Journal of Dermatology*.

[32] Leynaert B., Neukirch F., Demoly P., Bousquet J. (2000). Epidemiologic evidence for asthma and rhinitis comorbidity. *Journal of Allergy and Clinical Immunology*.

[33] Rönmark E., Ekerljung L., Lötvall J. (2012). Eczema among adults: prevalence, risk factors and relation to airway diseases. Results from a large-scale population survey in Sweden. *British Journal of Dermatology*.

[34] Vork K. L., Broadwin R. L., Blaisdell R. J. (2007). Developing asthma in childhood from exposure to secondhand tobacco smoke: insights from a meta-regression. *Environmental Health Perspectives*.

